# mRNA vaccine boosters and impaired immune system response in immune compromised individuals: a narrative review

**DOI:** 10.1007/s10238-023-01264-1

**Published:** 2024-01-27

**Authors:** Alberto Boretti

**Affiliations:** Melbourne Institute of Technology, The Argus, 288 La Trobe St, Melbourne, VIC 3000 Australia

**Keywords:** COVID-19, Vaccines, IgG 4 antibodies, CD4 + T cells, CD8 + T cells

## Abstract

Over the last 24 months, there has been growing evidence of a correlation between mRNA COVID-19 vaccine boosters and increased prevalence of COVID-19 infection and other pathologies. Recent works have added possible causation to correlation. mRNA vaccine boosters may impair immune system response in immune compromised individuals. Multiple doses of the mRNA COVID-19 vaccines may result in much higher levels of IgG 4 antibodies, or also impaired activation of CD4 + and CD8 + T cells. The opportunity for mRNA vaccine boosters to impair the immune system response needs careful consideration, as this impacts the cost-to-benefit ratio of the boosters’ practice.

## Introduction

The administration of mRNA vaccine boosters in individuals with impaired immune systems is an area of ongoing debate. The question of immunity to COVID-19 in immunocompromised individuals [1–4] is a critical and complex one, with reliable specific supporting information mostly missing, and a continuously evolving situation almost four years from the start of the outbreak. Immunocompromised individuals generally have weakened immune systems. Immunocompromised individuals may not mount as strong an immune response to vaccines compared to healthy individuals. This can affect the effectiveness of vaccination in preventing infection or severe disease. Booster doses have been recommended for immunocompromised individuals, to enhance and prolong immunity. Immunocompromised individuals may be at a higher risk of breakthrough infections, where they contract COVID-19 despite being fully vaccinated. The severity of breakthrough infections can also vary. Some immunocompromised individuals may not produce as many antibodies in response to the virus or the vaccine. Antibodies play a crucial role in neutralizing the virus and preventing infection. T-cell responses, which are another aspect of the immune system, might still provide some level of protection in immunocompromised individuals, even if antibody responses are limited. Regular monitoring of the immune status of immunocompromised individuals is crucial. However, this aspect is often overlooked. There is not reliable specific information available on the long-term evolution of immunity in immune-impaired populations over the four years following exposure to the SARS-CoV-2 virus, which has also undergone dramatic evolutions during these years progressing toward more infectious but less lethal variants. Long-term studies on the durability and evolution of immunity, especially in immunocompromised individuals, are missing.

There are concerns about the frequent use of COVID-19 booster shots, in immunocompromised individuals. There are some apprehensions associated with their frequent administration. There is a need to balance the benefits of boosting immunity against potential drawbacks, including waning vaccine effectiveness and the risk of diminishing immune response over time. The emergence of new variants adds complexity to booster strategies. Vaccines need to be updated to provide effective protection against new variants, and the frequency of booster doses could be influenced by the evolving virus landscape. Continuous monitoring is essential to assess the long-term safety of booster shots, especially in specific populations. There is a lack of reliable information inferred from long-term studies to evaluate the safety and efficacy of repeated booster doses in risk populations, with a virus also continuously evolving. The concept of immune exhaustion [5–9], deserve discussion in the context of frequent booster doses. It relates to concerns about the potential impact on the immune system over time. Immune exhaustion typically refers to a state where the immune response becomes less effective or diminished due to repeated stimulation. There is a lack of reliable information inferred from long-term studies to evaluate immune exhaustion caused by COVID-19 boosters in immunocompromised patients. The phenomenon of immune evasion [10–14], where certain viral variants partially or completely evade the immune response elicited by prior infection or vaccination, has also been observed with several SARS-CoV-2 variants. The impact of immune evasion varies across individuals. Individuals with impaired immunity may have a compromised ability to mount a robust immune response against the virus. The immune evasion by certain variants could potentially lead to increased susceptibility to reinfection or breakthrough infections in immunocompromised individuals. Some variants of concern have shown reduced susceptibility to neutralization by antibodies produced in response to vaccination or natural infection. This impact the effectiveness of vaccines in preventing infection or reducing the severity of illness. Immunocompromised individuals, who may already have a weaker immune response to vaccination, might be more susceptible to breakthrough infections with variants exhibiting immune evasion. Booster doses have been recommended to enhance and prolong immunity, especially in the face of emerging variants. However, the efficacy and safety of boosters which is influenced by the specific characteristics of the variants and the individual's immune status is not properly monitored. Even if a variant partially evades immunity, the severity of the infection can still vary. The immune system, even if not fully preventing infection, may still contribute to reducing the severity of the disease. As SARS-CoV-2 continues to evolve, and new variants emerge, the virus may adapt to selective pressures, including immune responses, leading to changes in its ability to evade the immune system. Variability exists among individuals in terms of immune responses. Even within the same population, some individuals may mount stronger immune responses than others, and this can influence susceptibility to variants. There is a lack of reliable information to evaluate immune exhaustion and evasion in immunocompromised patients.

The continuous mutation and emergence of new variants of the SARS-CoV-2 virus present additional challenges, particularly for immunocompromised individuals [15–19]. Some variants of SARS-CoV-2 have been classified as "Variants of Concern" due to their potential impacts on transmissibility, severity of illness, and immune evasion. These variants can pose challenges to public health efforts, including vaccination strategies. Variants may exhibit changes in key viral proteins, such as the spike protein, which can impact the virus's interaction with the immune system. This could result in partial or complete evasion of immunity generated through previous infection or vaccination. Some variants have shown reduced susceptibility to neutralization by antibodies elicited by existing vaccines. While booster doses have been recommended to enhance and extend immunity, especially in the face of emerging variants, this recommendation is not based on proven efficacy, and the side effects have been neglected. Certain variants have demonstrated increased transmissibility. This increased transmission can pose a higher risk to vulnerable populations, including immunocompromised individuals. Immunocompromised individuals may be at a higher risk of persistent viral infection, providing the virus with an extended opportunity for replication and evolution. This environment could potentially contribute to the emergence of new variants. Immunocompromised individuals may exhibit a range of immune responses, depending on the specific nature of their immunosuppression. Some individuals may have a diminished ability to mount an effective immune response against both the original virus and its variants.

Aim of this narrative review is to discuss if mRNA vaccine boosters impair immune system response in immunocompromised individuals.

## Method

A literature review is performed by using the google scholar database.

## Results

Over the last 18 months, there has been growing evidence of a correlation between the increased prevalence of COVID-19 infection and other pathologies, and the repetition of mRNA COVID-19 vaccine boosters [20, 21]. Recent works such as [22], and [23] are now evidencing causation to add to correlation, as multiple doses of the mRNA COVID-19 vaccines may result in much higher levels of IgG 4 antibodies, or also impaired activation of CD4 + and CD8 + T cells.

As outlined in [24], Undesirable outcomes observed after vaccination may be associated with the proinflammatory properties of the lipid nanoparticles or the delivered mRNA in the vaccine formulation. Additionally, these effects could be influenced by the distinctive characteristics, expression patterns, binding profiles, and proinflammatory tendencies of the generated antigens, particularly the spike (S) protein and its subunits/peptide fragments, within human tissues or organs.

A summary of negative aspects of repeated mRNA COVID-19 vaccine boosters is proposed in [25]. According to [26], already in 2021, the immune function was better among unvaccinated individuals than vaccinated individuals 8 months after the administration of two doses of the mRNA COVID-19 vaccine. The EU Medicine Agency warned in 2022 that frequent COVID-19 boosters could be counterproductive adversely impacting the immune system response [27]. A 4th dose of mRNA COVID-19 vaccine was mentioned in [28] to produce very little protection against being infected by the Omnicron variant.

According to [29], reduced cellular immunity may be the unexpected consequence of having replaced uracil in the genetic code with N1-methyl pseudo uridine, with activation of regulatory T cells caused by the modified protein. The spike protein on exosomes may persist for more than 4 months [30]. The mRNA of the COVID-19 vaccine was never supposed to only act in the place of injection in the muscle, but rather in the lymph nodes and the spleen [31–44]. This is central to explaining the many side effects such as [45–56], which include the unintended consequences on the immune system. The lipid nanoparticles encapsulating the mRNA of the COVID-19 vaccines build up in the liver, spleen, adrenal glands, and ovaries. The lipid nanoparticle component is highly inflammatory [57]. Cells that are primed to produce the spike protein are damaged [58]. Spike proteins in the bloodstream damage endothelial cells [59]. SARS-CoV-2 spike protein antibodies may have unintended consequences on other spike proteins [60]. The residual immune memory of the original SARS-CoV-2 virus may prevent efficacy against variants [61]. Signs of reduced immune system response have been many [62–64].

Reference [64] provide evidence suggesting that vaccination can significantly disrupt type I interferon signaling, leading to various adverse effects on human health. When immune cells take up vaccine nanoparticles, they release numerous exosomes into circulation, carrying the spike protein and critical microRNAs. These exosomes trigger signaling responses in recipient cells at distant locations. The findings also indicate potentially significant disruptions in the regulatory control of protein synthesis and cancer surveillance. These disruptions may be linked to neurodegenerative diseases, myocarditis, immune thrombocytopenia, Bell's palsy, liver disease, impaired adaptive immunity, compromised DNA damage response, and the initiation of tumorigenesis. This hypothesis is supported by evidence from the VAERS database.

Multiple doses of the mRNA COVID-19 vaccines lead to higher levels of IgG 4 antibodies [22]. Immunoglobulins are proteins produced by specialized white blood cells called B cells. They play a critical role in the immune response by recognizing and binding to specific foreign substances, such as viruses, bacteria, or other antigens. IgG4 is the least abundant subclass of IgG in the blood, accounting for only a small portion of total IgG antibodies. IgG4 antibodies are typically involved in immune responses associated with chronic inflammation and allergic reactions. One unique characteristic of IgG4 antibodies is their ability to undergo a process called “Fab arm exchange” [65, 66]. This process involves swapping one of the antigen-binding regions (Fab arms) with another IgG4 molecule, resulting in the formation of antibodies with two different specificities. This Fab arm exchange can reduce the overall effectiveness of IgG4 antibodies in certain immune responses. IgG4 antibodies are often associated with immune-mediated conditions. IgG4 antibodies serve various functions in the immune system, and their roles are still being studied and understood by researchers. Up to a certain level, these IgG 4 provide a protective effect [67]. Above a certain level, IgG 4 makes the immune system excessively susceptible to the COVID-19 spike protein [22]. Higher levels of IgG 4 were previously associated with patients who perished of COVID-19 infection [68, 69]. According to [22], Increased IgG4 synthesis due to repeated mRNA vaccine boosters may cause autoimmune diseases, and promote cancer growth and autoimmune myocarditis in susceptible individuals [22].

Multiple doses of the mRNA COVID-19 vaccines also produce impaired activation of CD4 + and CD8 + T cells [23]. The animal model of [23] then compared the humoral and cellular immune responses of an extended course of recombinant receptor binding domain (RBD) vaccine boosters. Multiple vaccine boosters after the conventional vaccination course significantly decreased RBD-specific antibody titers and serum-neutralizing efficacy against the Delta and Omicron variants, profoundly impaired CD4 + and CD8 + T cell activation, and increased PD-1 and LAG-3 expressions in these T cells. Impaired activation of CD4 + and CD8 + T cells can have various consequences on the immune response and overall immune system function [70–72].

CD4 + T cells play a crucial role in orchestrating and regulating immune responses [73]. They help activate other immune cells, such as B cells and CD8 + T cells, and coordinate the overall immune response against infections or pathogens. Impaired CD4 + T cell activation can lead to a weakened immune response [74], making it more challenging for the body to fight off infections effectively. CD4 + T cells are involved in providing help to B cells, which are responsible for producing antibodies. Impaired CD4 + T cell activation can hinder this collaboration, resulting in reduced antibody production. This can compromise the ability to mount an effective humoral immune response against pathogens.

CD8 + T cells are a vital component of cell-mediated immunity [75, 76]. They are responsible for recognizing and eliminating infected or abnormal cells, including virus-infected cells or cancer cells. Impaired activation of CD8 + T cells can compromise the ability to target and destroy these abnormal cells, allowing infections or tumor growth to persist. With impaired CD4 + and CD8 + T cell activation, the immune system becomes less efficient in responding to infections [77, 78]. This can lead to an increased susceptibility to various infectious agents, including bacteria, viruses, fungi, and parasites. CD4 + and CD8 + T cells play a role in regulating inflammation and preventing excessive immune activation. Impairment in their activation can disrupt this regulation, leading to prolonged or chronic inflammation. Chronic inflammation is associated with various autoimmune diseases and other chronic conditions.

CD4 + T cells are particularly crucial in providing immune defense against opportunistic infections, which are caused by pathogens that typically do not cause disease in individuals with a healthy immune system [79, 80]. Impaired CD4 + T cell activation, as seen in conditions like HIV/AIDS, can result in a higher risk of opportunistic infections. For example, mycosis fungoides, which is a type of cutaneous T-cell non-Hodgkin's lymphoma, is characterized by the abnormal accumulation of CD4 + T cells in the skin [81–83]. While impaired activation of CD4 + and CD8 + T cells is not considered the direct cause of mycosis fungoides, for which the exact cause is not fully understood, it is believed to play a role in the pathogenesis of the disease. The work [23] confirms that extended vaccination with RBD boosters overturns the protective immune memories by promoting adaptive immune tolerance. The work [23] casts serious doubt about the protective efficacy of mRNA COVID-19 boosters also bearing significant potential adverse effects.

As shown in [84], a second monovalent mRNA booster may cause supplementary protection against symptomatic Omicron BA.2 or BA.4/5 infections, about a first booster given 6–7 months earlier. Nevertheless, the improvement in protection presented by a second booster was less than the protection observed with a first booster, at the same time points since these booster doses. Additionally, prior infection, in a vaccinated population, presented higher levels and long-lasting protection against symptomatic Omicron BA.2 or BA.4/5 infections. Natural immunity is far superior to that achieved by vaccination, as well-known since 2021 [85–88].

Given the emergence of post-COVID-19 vaccination syndrome [89], Ref. [90] notices that given the much-reduced lethality of the virus, since the times the Omnicron variant emerged, the practice of vaccine boosters may be counterproductive in the healthy population as well as patients with Autoimmune Diseases, or Cardiac Issues.

## Discussion

Monoclonal antibody treatment [91], which is a possible alternative to vaccination, has been marginally explored in immunocompromised individuals for several reasons, as it usually serves a different purpose than immunization. Monoclonal antibody treatments could be used both for prophylaxis (prevention) and as a therapeutic option for individuals who have already been infected making them a valuable tool for both preventing and treating COVID-19 in immunocompromised individuals. Monoclonal antibodies can be administered early in the course of infection, ideally within the first few days after symptoms develop. This early intervention can help reduce the severity of the illness and prevent progression to severe disease, hospitalization, or death. For individuals who may not mount a robust immune response to natural infection or vaccination, monoclonal antibodies can provide an additional layer of protection by offering ready-made antibodies targeting the virus. Monoclonal antibodies designed to specifically target the SARS-CoV-2 virus may bind to the virus and neutralize its ability to infect cells. Immunocompromised individuals often face a higher risk of severe outcomes if they contract COVID-19. Monoclonal antibodies can help mitigate this risk by providing immediate passive immunity.

## Conclusion

A considerable body of evidence indicates a correlation, and some recent studies even suggest causation, highlighting the potential for mRNA COVID-19 boosters to have adverse effects on the immune system. This is particularly relevant in the case of immunocompromised individuals, where the overall cost-to-benefit ratio may lean toward the negative. A comprehensive, evidence-based assessment is essential to promptly evaluate the implications of continuous COVID-19 vaccine booster use for this specific population. Given the decreased severity of the virus, as acknowledged in various jurisdictions, there are legitimate concerns about the frequent administration of boosters in immunocompromised patients, raising questions about whether this practice may be causing more harm than benefit.

## Declarations

### Trial registration

Not applicable, because this article does not contain any clinical trials.
